# Collaboration and distances between German immunological institutes – a trend analysis

**DOI:** 10.1186/1747-5333-1-6

**Published:** 2006-06-14

**Authors:** Frank Havemann, Michael Heinz, Hildrun Kretschmer

**Affiliations:** 1 Department of Library and Information Science, Humboldt University Berlin, Dorotheenstr. 26, D-10099 Berlin, Germany

## Abstract

**Background:**

The hypothesis that distance matters but that in recent years geographical proximity has become less important for research collaboration was tested. We have chosen a sample–authors at German immunological institutes–that is relatively homogeneous with regard to research field, language and culture, which beside distance are other possible factors influencing the willingness to co-operate. We analyse yearly distributions of co-authorship links between institutes and compare them with the yearly distributions of distances of all institutes producing papers in journals indexed in the Science Citation Index, editions 1992 till 2002. We weight both types of distributions properly with paper numbers.

**Results:**

One interesting result is that place matters but if a researcher has to leave the home town to find a collaborator distance does not matter any longer. This result holds for all years considered, but is statistically most significant in 2002. The tendency to leave the own town for collaborators has slightly increased in the sample. In addition, yearly productivity distributions of institutes have been found to be lognormal.

**Conclusion:**

The Internet did not change much the collaboration patterns between German immunological institutes.

## Background

The global and secular tendency to more and more collaboration in scientific research has been demonstrated in many scientometric and other studies. To establish and maintain collaboration links can be more or less easy, depending on scientific, cultural, political, and geographical barriers which have to be overcome. In recent years some of these barriers have been lowered. The Iron Curtain does not longer exist, air flights became less costly and the Internet has made telecommunication easy, cheap and fast. We have tested the hypothesis that in recent years geographical proximity has become less important for establishing research collaboration. For this test we chose a sample of institutions where all other barriers for collaboration mentioned above are nearly absent. We analysed the collaboration between German institutions of immunology.

A similar study was made by Sylvan Katz [1]. He observed that in Australia, Canada, and UK the numbers of papers which members of two universities published together decreased exponentially with their distance. Smith and Katz [2] found, that UK "life sciences showed the largest change in their geographical collaboration pattern, the average distance between collaborating institutions increasing over the time period [1980–1994]. The pattern of geographical collaborations in the natural sciences remained quite constant over time." Liang Liming [3] obtained for inter-regional co-operation in China that geographical proximity is an important factor. Nagpaul [4] studied international research collaboration and concluded that geographical proximity has greater positive impact than thematic proximity and socio-economic proximity.

## Methods

### Data

The papers taken into account for this study are drawn from the Science Citation Index (SCI). We analysed the publications of German research institutions which are listed on the website of the German Society for Immunology [5]. We took all records in the SCI CD-ROM editions 1992–1999 and 2002 into account, which had at least one author affiliated with one of 80 institutes mentioned on the list. We used a search strategy (developed with 2002 data) to avoid tedious search for address variants. We found a monotonically increasing number of institutions in SCI (1992: *n *= 54, 2002: *n *= 73).

### Definitions

It is not sufficient (although often done [1,6]) to plot the distribution of distances of co-operating institutes to prove that distance matters. You have to compare this distribution with the distribution of distances between all pairs of institutes in the sample (here called Institutional Distance Distribution or IDD). In our case this IDD is determined by the geographical scattering of immunological institutes in Germany. Moreover, we felt that the intensity of collaboration should be taken into account, too. Therefore we weighted the distance distribution of co-operating institutes with the number of co-authored papers. Doing this we disregard that an increasing number of papers were produced together with partners who do not belong to our sample of German immunological institutes (cf. Conclusions, below). Because we found only three papers where authors from more than two sample institutes were involved, nearly all papers considered appear only once as a weight of the co-authorship weighted IDD. If triple collaboration within the sample would be more frequent we should have measured productivity of institutes by fractionally counted paper numbers [7].

Then the problem arises, to which distance distribution this Co-authorship Weighted IDD should be compared. We propose to use a distribution of distances weighted with the geometric mean of paper numbers of both institutions. Our argument for this choice is twofold. First, the productivity (in number of papers) of institutes is lognormally distributed (see below). Thus, the geometric mean has to be used and not the arithmetic one. This has the advantage, that institutes with no papers in a year are automatically excluded. Second, Salton's (cosine) measure applied to collaboration of two institutes with paper numbers *a *and *b*, respectively, and *c *co-authored papers is *S *= *c*/. Thus, we compare a distance distribution weighted with the numerator of Salton's measure with one weighted with its denominator. We also check whether the Salton Index of co-operation does correlate with distance. This Index reflects both actual and potential collaborations, as we have required above.

Alternatively, one can also use the geometric mean of numbers of all collaborative papers of both institutes to weight the comparison distribution. This number more sharply reflects the capacity of two institutes for mutual co-operation.

The following definitions of distance distributions are used in this paper:

**Definition 1 (IDD) ***The Institutional Distance Distribution (IDD) is the distribution of distances between German immunological institutes on streets and highways (taken from a route planner)*.

**Definition 2 (Collaboration IDD) ***Collaboration IDDs are (annual) distributions of distances between pairs of institutes co-authoring papers*.

**Definition 3 (Co-authorship Weighted IDD) ***Co-authorship Weighted IDDs are (annual) distributions of distances between pairs of institutes co-authoring papers weighted with number of co-authored papers*.

**Definition 4 (Productivity Weighted IDD) ***Productivity Weighted IDDs are (annual) distributions of distances of pairs of all German immunological institutes weighted with the geometric mean of paper numbers of both institutions*.

## Results and discussion

### Does distance matter?

First we compared unweighted real co-authorship links with all possible links between the institutes in the sample. We made a Kolmogorov-Smirnov Goodness-of-Fit test (KS-test) to check whether the annual Collaboration IDDs (cf. Definitions above) behave like random samples from the IDD. The answer is *No*. The test values are between 2.64 (1992) and 3.69 (1996) and thus exceed the critical value 1.63 (99% level). Here we can reject the Null hypothesis that collaboration links are independent from distance with less than 1 percent failure probability. Short distances are preferred.

Then we made a comparison of co-authorship weighted links with paper weighted possible links. We asked: Do the annual Co-authorship Weighted IDDs behave like random samples from annual Productivity Weighted IDDs? The answer was negative, too. KS-Test values here are between 2.56 (1992) and 3.41 (2002). Short distances are clearly preferred, again.

**Figure 1:**
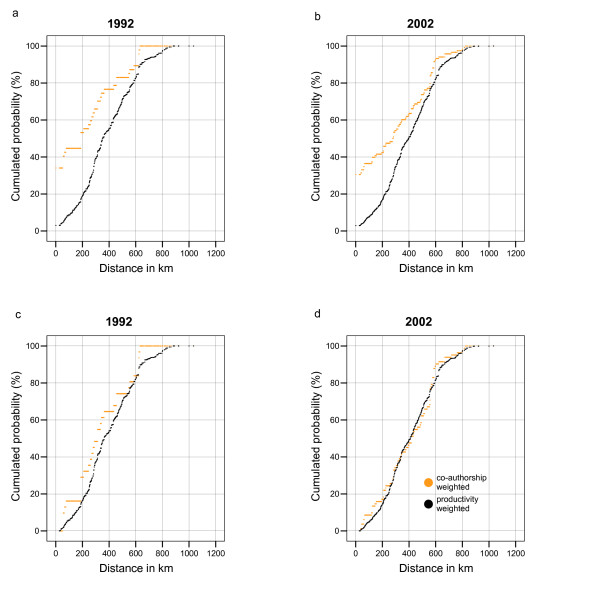
**Weighted distribution functions**. Weighted distribution functions: a) and b) Links within towns included. c) and d) Without links within towns.

Next we tested the hypothesis that very short distances are preferred. For this reason we omitted collaborations between institutes within the same town. Now nearly all KS tests suggest that the Null hypothesis should not be rejected if we demand less than 5 percent failure probability (critical value: 1.36). All but one of the KS test values in unweighted case were found between 0.82 (1998) and 1.22 (1995). In weighted case all but one of the test values lay between 0.77 (2002) and 1.39 (1999). In both cases, only in 1996 the test values are above the critical value 1.63 (1 percent failure probability; unweighted: 1.64, weighted: 1.67). That means, when in-town links are omitted, annual Co-authorship Weighted IDDs behave like random samples from annual Productivity Weighted IDDs. The same is true for the unweighted case. Figure 1 shows plots of different distribution functions for the first and the last year considered. If the observed distribution function (pale curve) is always near to the comparison function (black curve) then the Null hypothesis cannot be rejected. We also checked whether the Salton Index of co-operation does decrease with distance. There is nearly no correlation. If zero distances (partners in the same town) are omitted it disappears totally.

"Physical connectivity and degrees of presence matter, even in an age of electronic publication, online communities and digital networks" as Blaise Cronin [8] said recently. Place matters, but if a researcher has to leave the home town to find a collaborator distance does not matter any longer. This result holds for all years considered, but is statistically most significant in 2002.

### Does geographical proximity become less important?

**Figure 2:**
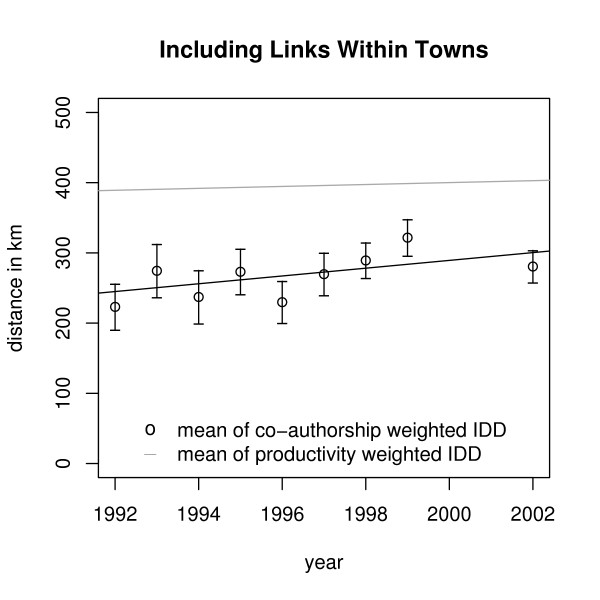
**Trends of means of IDDs**. Trends of means of IDDs including links within towns.

**Figure 3:**
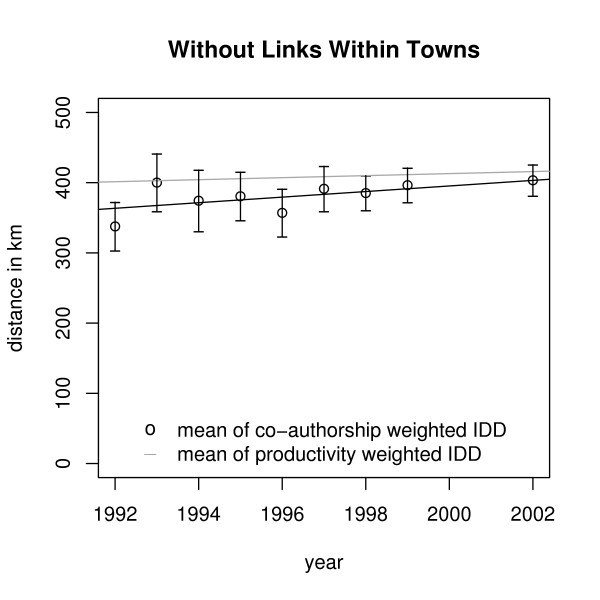
**Trends of means of IDDs**. Trends of means of IDDs without links within towns.

We found a trend of means (and medians) of annual Co-authorship Weighted IDDs toward longer distances. Including links within towns the mean increases by 5.5 ± 3.2 km p.a., without links within towns the mean increases by 4.0 ± 3.4 km p.a. But there is also a (weaker) trend of means (and medians) of annual Productivity Weighted IDDs: Including links within towns the mean is increasing by 1.37 km p.a., without links within towns by 1.44 km p.a. (Figures 2 and 3).

Thus, we subtracted the trends of our comparison distributions and got a mean increasing by 4.2 ± 3.2 km p.a. including links within towns. When only collaborations between institutes at different places are considered, we got 2.5 ± 3.2 km p.a. That means, there is no indication of a significant trend. So, a little more willingness for leaving the place can be stated but the choice of partners outside the town remains independent of distance in agreement with the result reported above.

### Collaboration indicators

The number of authors per paper was about 5 till 1996, about 6 later. The Collaboration Coefficient [9] increased from 0.79 in 1992 to 0.84 in 2002. We found typically two addresses per paper till 1994, about three later. The number of papers produced jointly by (exactly) two German immunological institutions (and, possibly, other foreign or German non-immunological ones) increased by 7.2 papers per year on average (*R*^2^= .96) from 47 in 1992 to 118 in 2002. That is about 3 percent of all their papers in SCI till 1999 and 4 percent in 2002.

International collaboration also grew in our sample. The share of papers with foreign collaboration partners increased from 28 percent in 1992 to 38 percent in 2002. The share of papers with partners from two or more other countries (multilateral cooperation) increased from 5 percent in 1992 to 10 percent in 2002.

### Productivity of institutes

In every year the productivity of institutes is lognormally distributed–if institutes without papers in SCI are excluded. The KS-tests are passed with test-values from *D* = 0.53 in 1994 to *D* = 0.76 in 1996. The geometric means are 12, ..., 15 papers till 1997, 17 or 18 after 1997. Standard deviations of log(productivity) lay between 0.5 and 0.6.

## Conclusion

The Internet did not change much the collaboration patterns between German immunological institutes. Before and after it became the main communication tool of scientists these institutes did not much care about geographical distance, when selecting their German co-operation partners outside their home town. Partners in the same town collaborate at higher rate, also before and in the Internet era. The hypothesis that in recent years geographical proximity has become less important for establishing research collaboration within a country for the case of German immunological institutes can only be confirmed in a weak sense. The willingness to leave the home town has increased slightly.

One referee wondered whether the picture obtained would be different if the analysed population of institutes were divided along the former border between East and West Germany. We did not analyse this because the German unification and the transformation process of the East German science system after the fall of the Wall led to more and more East-West collaboration (partly caused by Western scientists getting positions in the East) [10]. Thus, our main result of distance independency cannot be changed by a East-West division of the population of institutes.

The same referee pointed at the issue of status barriers which can hinder collaboration in addition to the barriers mentioned by us in the background section. The status of an institution is indicated by its productivity (and also by its visibility). It would be of interest to look at the geographical distribution of institutional productivity. We take this distribution into account by weighting our comparison distribution (the IDD) with paper numbers of collaborating institutes.

The increasing percentage of internationally co-authored papers is a well-known fact (cf., e.g., Figure 5–40 in *Science and Engineering Indicators 2004 *[11]). We cannot generalise the results of our intra-national study to international collaboration which is influenced by geographical proximity and by some other factors (cf. p. 264 in Reference [12]).

For a finer analysis papers of institutions should be counted fractionally to assure that the trend to more collaboration does not inflect the results [7] but we suppose that the effect will be small because counting fractionally lowers both the weights of the Co-authorship IDDs and the weights of the comparison distributions.

## Declaration of competing interests

The author(s) declare that they have no competing interests.

## Authors' contributions

H. K. had the idea for this study and delivered results of a pilot study. The search strategy was developed by M. H. and tested by F. H. Calculations and figures were done by M. H. The first draft was written by F. H. He also made some figures and the revised version. We all discussed this version.
